# Di-μ-chlorido-bis­[bis­(η^2^-cyclo­octene)iridium(I)]

**DOI:** 10.1107/S1600536808007216

**Published:** 2008-03-29

**Authors:** Tsuneaki Yamagata, Koji Nakajima, Kenji Arimitsu, Aika Iseki, Kazuhide Tani

**Affiliations:** a Department of Chemistry, Graduate School of Engineering Science, Osaka University, Machikaneyama, 1-3, Toyonaka, Osaka 560-8531, Japan; b Higashiosaka College, Nishitutumi Gakuen-chou 3-1-1, Higashiosaka, Osaka 577-8567, Japan

## Abstract

The title complex, [Ir_2_(μ-Cl)_2_(C_8_H_14_)_4_], has a dinuclear structure with bridging Cl atoms, a hinge angle of 179.44 (7)° between the two IrCl_2_ planes, and an Ir⋯Ir distance of 3.7254 (3) Å. Regarding the coordinating C=C bonds as occupying a single coordination site each, the geometry around each Ir atom is square-planar.

## Related literature

For related literature, see: Cotton *et al.* (1986 ▶); De Ridder & Imhoff (1994 ▶); Dorta *et al.* (1997 ▶); Herde *et al.* (1974 ▶); Pettinari *et al.* (2002 ▶); Tani *et al.* (1985 ▶, 1995 ▶); Yamagata *et al.* (1997 ▶, 2007*a* ▶,*b* ▶).
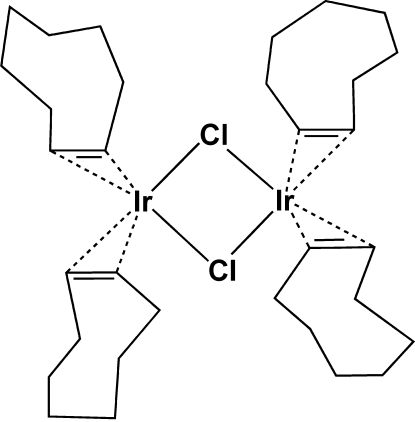


## Experimental

### 

#### Crystal data

[Ir_2_Cl_2_(C_8_H_14_)_4_]
*M* *_r_* = 896.07
Monoclinic, 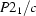

*a* = 12.3410 (5) Å
*b* = 10.7713 (3) Å
*c* = 23.6450 (6) Å
β = 91.7873 (13)°
*V* = 3141.57 (16) Å^3^
*Z* = 4
Mo *K*α radiation
μ = 8.65 mm^−1^
*T* = 100 (1) K
0.19 × 0.09 × 0.05 mm

#### Data collection

Rigaku R-AXIS RAPID Imaging Plate diffractometer
Absorption correction: numerical (*NUMABS*; Higashi, 1999 ▶) *T*_min_ = 0.585, *T*_max_ = 0.830
39295 measured reflections
7172 independent reflections
6321 reflections with *I* > 2σ(*I*)
*R*_int_ = 0.055

#### Refinement

*R*[*F* ^2^ > 2σ(*F* ^2^)] = 0.033
*wR*(*F* ^2^) = 0.068
*S* = 1.08
7172 reflections
349 parameters
5 restraints
H atoms treated by a mixture of independent and constrained refinement
Δρ_max_ = 2.46 e Å^−3^
Δρ_min_ = −1.67 e Å^−3^

### 

Data collection: *RAPID-AUTO* (Rigaku, 1998 ▶); cell refinement: *RAPID-AUTO*; data reduction: *TEXSAN* (Rigaku/MSC, 2004 ▶); program(s) used to solve structure: *SIR97* (Altomare *et al.*, 1999 ▶); program(s) used to refine structure: *SHELXL97* (Sheldrick, 2008 ▶); molecular graphics: *ORTEP-3* (Farrugia, 1997 ▶); software used to prepare material for publication: *SHELXL97*.

## Supplementary Material

Crystal structure: contains datablocks global, I. DOI: 10.1107/S1600536808007216/cf2181sup1.cif

Structure factors: contains datablocks I. DOI: 10.1107/S1600536808007216/cf2181Isup2.hkl

Additional supplementary materials:  crystallographic information; 3D view; checkCIF report

## Figures and Tables

**Table:** 

Ir1—C10	2.113 (5)
Ir1—C2	2.123 (5)
Ir1—C1	2.138 (6)
Ir1—C9	2.139 (5)
Ir1—Cl1	2.3980 (12)
Ir1—Cl2	2.4188 (12)
Ir1⋯Ir2	3.7254 (3)
Ir2—C26	2.117 (5)
Ir2—C18	2.117 (5)
Ir2—C25	2.139 (5)
Ir2—C17	2.153 (5)
Ir2—Cl1	2.4036 (12)
Ir2—Cl2	2.4203 (12)

**Table:** 

Cl1—Ir1—Cl2	78.84 (4)
Cl1—Ir2—Cl2	78.70 (4)
Ir1—Cl1—Ir2	101.77 (5)
Ir1—Cl2—Ir2	100.69 (4)

**Fig. 1.**
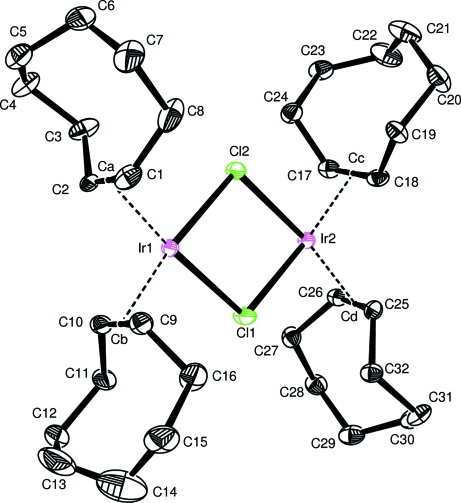
The molecular structure, showing the atom labelling scheme. Displacement ellipsoids are drawn at the 50% probability level. All H atoms are omitted. Ca, Cb, Cc and Cd are the centroids of the coordinated C?C bonds.

## supplementary crystallographic information

### Comment

1,5-Cyclooctadiene (cod) or cyclooctene (coe) complexes of rhodium(I) and iridium(I) with general formulae [MX(cod)]_2_ or [MX(coe)_2_]_2_ (*M* = Ir^I^ or Rh^I^; *X* = Cl or Br or I) have been used as key starting compounds for various rhodium and iridium complexes. For example, an excellent asymmetric catalyst precursor, [Rh{(*R*)-binap}_2_]ClO_4_ (Tani *et al.*, 1985) or [Ir(µ-Cl){(*R*)-binap}]_2_ (Yamagata *et al.*, 1997; Dorta, *et al.*, 1997; Tani *et al.*, 1995), has been prepared from the reaction of [RhCl(cod)]_2_ or the title compound, [Ir(µ-Cl)(C_8_H_14_)_2_]_2_ (I), respectively, with (*R*)-BINAP {(*R*)-(+)-2,2'-bis(diphenylphosphino)-1–1'-binaphthyl}. The X-ray structure analyses of a series of the cod complexes have been reported. However, the crystal structures of the coe complexes have not been determined. Thus, we report here the preparation and the crystal structure of the title complex (I), which reveals also a dinuclear iridium complex (Fig. 1). The coordination geometry defined by bridging chlorine atoms and centroids of double bonds around Ir1 and Ir2 is essentially square planar. The hinge angle ((Ir1 Cl1 Cl2)/(Ir2 Cl1 Cl2) = 179.44 (7)°) is nearly 180° and the Ir···Ir distance is 3.7254 (3) Å. All the cod complexes have an analogous halogen-bridged dinuclear structure. The rhodium complex, [Rh(µ-Cl)(cod)]_2_ (De Ridder & Imhoff, 1994), showed an almost planar structure (the hinge angle is 169.1 (3)°), whereas [Ir(µ-I)(cod)]_2_ (Yamagata, *et al.*, 2007*a*), [Ir(µ-Br)(cod)]_2_ (Yamagata *et al.*, 2007*b*), [Ir(µ-Cl)(cod)]_2_ (Cotton *et al.*, 1986), and [Rh(µ-Br)(cod)]_2_ (Pettinari *et al.*, 2002) show bent structures, with hinge angles of 95.26 (1)°, 101.58 (3)°, 109.4 (3)°, and 148.7 (3)°, respectively. The M···*M* distances in [Ir(µ-I)(cod)]_2_, [Ir(µ-Br)(cod)]_2_, [Ir(µ-Cl)(cod)]_2_, and [Rh(µ-Br)(cod)]_2_ are 2.9228 (6) Å, 2.9034 (5) Å, 2.910 (1) Å, and 3.565 Å, respectively. The degree of bending is Ir > Rh and I > Br > Cl. By replacing a cod ligand with two coe ligands the coordination geometries change considerably.

### Experimental

The title compound was prepared according to a modified literature method (Herde *et al.*, 1974). All manipulations of air-sensitive materials were performed under argon using standard Schlenk and vacuum techniques (8 *x* 10 ^-2^ Torr). IrCl_3_.3H_2_O (2.0 g, 5.67 mmol) was placed in a 100 ml round-bottomed flask. To this were added water (12 ml) and 2-propanol (22 ml). After addition of cyclooctene (3.5 ml), the reaction mixture was refluxed at 353 K for 3 hr. The colour of the reaction mixture turned from dark red to orange-yellow and a yellow suspension was formed. The reaction mixture was cooled to ambient temperature. The yellow precipitate was collected, washed with ice-cooled methanol (20 ml *x* 2), and then dried *in vacuo* to afford 2.04 g (2.28 mmol, 80%) of the pure product. NMR (270.05 MHz, CDCl_3_, 308 K, δ, p.p.m.): 2.17–2.08 (m, 4H), 1.92–1.85 (m, 2H), 1.67–1.60 (m, 2H), 1.53–1.32 (m, 6H). Orange single crystals for X-ray analysis were grown from a solution in THF under argon.

### Refinement

The C12—C13 and C13—C14 bond lengths and C12···C14 distance were restrained to 1.53 (2) Å and 2.50 (2) Å, respectively. All H atoms were located in a difference Fourier map. H atoms except the olefinic H atoms were included with a riding model (C—H = 0.99 Å, *U*_iso_(H) = 1.2*U*_eq_(C)). The atomic coordinates of the olefinic H atoms were refined, with C1—H1 and C2—H2 restrained to 0.95 (2) Å. The final difference Fourier map gave a maximum peak (2.463 e Å^-3^), which was present near the atom C13 (1.10 Å). The deepest hole of the final difference Fourier map was -1.668 e Å^-3^ (0.78 Å from Ir1).

### Crystal data

**Table:**

[Ir_2_Cl_2_(C_8_H_14_)_4_]	*F*_000_ = 1744
*M**_r_* = 896.07	*D*_x_ = 1.895 Mg m^−^^3^
Monoclinic, *P*2_1_/*c*	Mo *K*α radiation λ = 0.71069 Å
Hall symbol: -P 2ybc	Cell parameters from 78044 reflections
*a* = 12.3410 (5) Å	θ = 2.1–31.6º
*b* = 10.7713 (3) Å	µ = 8.65 mm^−^^1^
*c* = 23.6450 (6) Å	*T* = 100 (1) K
β = 91.7873 (13)º	Block, orange
*V* = 3141.57 (16) Å^3^	0.19 × 0.09 × 0.05 mm
*Z* = 4	

### Data collection

**Table:**

Rigaku R-AXIS RAPID Imaging Plate diffractometer	7172 independent reflections
Radiation source: normal-focus sealed tube	6321 reflections with *I* > 2σ(*I*)
Monochromator: graphite	*R*_int_ = 0.055
Detector resolution: 10.00 pixels mm^-1^	θ_max_ = 27.5º
*T* = 100(1) K	θ_min_ = 2.5º
ω scans	*h* = −16→15
Absorption correction: numerical(NUMABS; Higashi, 1999)	*k* = −13→13
*T*_min_ = 0.585, *T*_max_ = 0.830	*l* = −30→30
39295 measured reflections	

### Refinement

**Table:**

Refinement on *F*^2^	Secondary atom site location: difference Fourier map
Least-squares matrix: full	Hydrogen site location: inferred from neighbouring sites
*R*[*F*^2^ > 2σ(*F*^2^)] = 0.033	H atoms treated by a mixture of independent and constrained refinement
*wR*(*F*^2^) = 0.068	*w* = 1/[σ^2^(*F*_o_^2^) + (0.0182*P*)^2^ + 25.7043*P*] where *P* = (*F*_o_^2^ + 2*F*_c_^2^)/3
*S* = 1.08	(Δ/σ)_max_ = 0.001
7172 reflections	Δρ_max_ = 2.46 e Å^−^^3^
349 parameters	Δρ_min_ = −1.67 e Å^−^^3^
5 restraints	Extinction correction: none
Primary atom site location: structure-invariant direct methods	

### Special details

**Table:**

Experimental. Indexing was performed from 2 oscillations which were exposed for 500 s. The camera radiuswas 127.40 mm. Readout performed in the 0.100 mm pixel mode. #1 Phi= 90.0, chi=55.0, omega=50.0 to 230.0 with 3.0deg step #2 Phi=300.0, chi=40.0, omega=70.0 to 250.0 with 3.0deg stepA total of 120 images, corresponding to 360.0 °. osillation angles, were collected with 2 different goniometer setting. Exposure time was 100 s per degree. The camera radiuswas 127.40 mm. Readout performed in the 0.100 mm pixel mode.
Geometry. All e.s.d.'s (except the e.s.d. in the dihedral angle between two l.s. planes) are estimated using the full covariance matrix. The cell e.s.d.'s are taken into account individually in the estimation of e.s.d.'s in distances, angles and torsion angles; correlations between e.s.d.'s in cell parameters are only used when they are defined by crystal symmetry. An approximate (isotropic) treatment of cell e.s.d.'s is used for estimating e.s.d.'s involving l.s. planes. Least-squares planes (*x*,*y*,*z* in crystal coordinates) and deviations from them (* indicates atom used to define plane)2.1389 (0.0071) *x* + 3.6982 (0.0062) *y* + 21.6879 (0.0063) *z* = 3.5218 (0.0013)* 0.0000 (0.0000) Ir1 * 0.0000 (0.0000) Cl1 * 0.0000 (0.0000) Cl2 0.0181 (0.0020) Ir2Rms deviation of fitted atoms = 0.00002.0283 (0.0066) *x* + 3.7376 (0.0066) *y* + 21.7009 (0.0061) *z* = 3.4830 (0.0032)Angle to previous plane (with approximate e.s.d.) = 0.56 (0.07)* 0.0000 (0.0000) Ir2 * 0.0000 (0.0000) Cl1 * 0.0000 (0.0000) Cl2 0.0180 (0.0020) Ir1Rms deviation of fitted atoms = 0.0000
Refinement. Refinement of *F*^2^ against ALL reflections. The weighted *R*-factor *wR* and goodness of fit *S* are based on *F*^2^, conventional *R*-factors *R* are based on *F*, with *F* set to zero for negative *F*^2^. The threshold expression of *F*^2^ > σ(*F*^2^) is used only for calculating *R*-factors(gt) *etc*. and is not relevant to the choice of reflections for refinement. *R*-factors based on *F*^2^ are statistically about twice as large as those based on *F*, and *R*- factors based on ALL data will be even larger.

### Fractional atomic coordinates and isotropic or equivalent isotropic displacement parameters (Å^2^)

**Table:**

	*x*	*y*	*z*	*U*_iso_*/*U*_eq_	
Ir1	0.230075 (15)	0.075614 (17)	0.126802 (8)	0.01383 (5)	
Ir2	0.509155 (15)	−0.055152 (17)	0.122410 (8)	0.01382 (5)	
Cl1	0.40939 (10)	0.13086 (12)	0.09970 (6)	0.0223 (3)	
Cl2	0.32842 (10)	−0.11317 (11)	0.14929 (6)	0.0189 (2)	
C1	0.0736 (4)	−0.0096 (6)	0.1264 (3)	0.0243 (12)	
H1	0.025 (4)	0.031 (6)	0.101 (2)	0.029*	
C2	0.1000 (4)	0.0349 (5)	0.1804 (2)	0.0191 (11)	
H2	0.064 (5)	0.109 (4)	0.192 (3)	0.023*	
C3	0.1213 (5)	−0.0501 (6)	0.2304 (2)	0.0277 (13)	
H3A	0.1752	−0.0113	0.2568	0.033*	
H3B	0.1518	−0.1296	0.2172	0.033*	
C4	0.0165 (5)	−0.0750 (6)	0.2613 (3)	0.0286 (13)	
H4A	−0.0035	0.0013	0.2818	0.034*	
H4B	0.0308	−0.1406	0.2899	0.034*	
C5	−0.0811 (5)	−0.1151 (6)	0.2238 (3)	0.0280 (13)	
H5A	−0.1426	−0.1325	0.2484	0.034*	
H5B	−0.1023	−0.0446	0.1990	0.034*	
C6	−0.0632 (6)	−0.2276 (6)	0.1870 (3)	0.0376 (16)	
H6A	−0.1274	−0.2822	0.1895	0.045*	
H6B	−0.0002	−0.2739	0.2030	0.045*	
C7	−0.0436 (6)	−0.2035 (7)	0.1243 (3)	0.0421 (18)	
H7A	−0.1015	−0.1477	0.1093	0.050*	
H7B	−0.0507	−0.2832	0.1037	0.050*	
C8	0.0669 (6)	−0.1457 (7)	0.1110 (3)	0.0413 (18)	
H8A	0.1249	−0.1914	0.1321	0.050*	
H8B	0.0797	−0.1553	0.0701	0.050*	
C9	0.1533 (5)	0.2178 (5)	0.0768 (2)	0.0221 (11)	
H9	0.075 (6)	0.202 (6)	0.071 (3)	0.027*	
C10	0.1791 (5)	0.2624 (5)	0.1318 (3)	0.0217 (11)	
H10	0.120 (6)	0.270 (6)	0.154 (3)	0.026*	
C11	0.2647 (5)	0.3610 (5)	0.1432 (3)	0.0283 (13)	
H11A	0.3238	0.3510	0.1161	0.034*	
H11B	0.2961	0.3503	0.1819	0.034*	
C12	0.2155 (6)	0.4936 (6)	0.1370 (3)	0.0365 (16)	
H12A	0.2756	0.5543	0.1359	0.044*	
H12B	0.1738	0.5119	0.1711	0.044*	
C13	0.1382 (7)	0.5137 (7)	0.0825 (4)	0.067 (3)	
H13A	0.0751	0.4576	0.0855	0.081*	
H13B	0.1104	0.5999	0.0833	0.081*	
C14	0.1858 (9)	0.4931 (8)	0.0281 (4)	0.067 (3)	
H14A	0.2657	0.4956	0.0335	0.080*	
H14B	0.1649	0.5632	0.0030	0.080*	
C15	0.1567 (6)	0.3761 (7)	−0.0015 (3)	0.0381 (16)	
H15A	0.1782	0.3831	−0.0414	0.046*	
H15B	0.0770	0.3664	−0.0016	0.046*	
C16	0.2089 (6)	0.2587 (6)	0.0241 (3)	0.0327 (14)	
H16A	0.2051	0.1909	−0.0042	0.039*	
H16B	0.2863	0.2752	0.0335	0.039*	
C17	0.5778 (4)	−0.2077 (5)	0.1702 (2)	0.0178 (10)	
H17	0.641 (5)	−0.177 (6)	0.185 (3)	0.021*	
C18	0.5765 (5)	−0.2340 (5)	0.1113 (2)	0.0198 (11)	
H18	0.637 (6)	−0.223 (6)	0.093 (3)	0.024*	
C19	0.5016 (5)	−0.3272 (5)	0.0825 (2)	0.0221 (11)	
H19A	0.4916	−0.3038	0.0422	0.027*	
H19B	0.4299	−0.3228	0.1000	0.027*	
C20	0.5427 (6)	−0.4622 (6)	0.0858 (3)	0.0321 (14)	
H20A	0.5005	−0.5126	0.0579	0.039*	
H20B	0.6194	−0.4638	0.0748	0.039*	
C21	0.5347 (6)	−0.5229 (6)	0.1440 (3)	0.0385 (16)	
H21A	0.5444	−0.6136	0.1395	0.046*	
H21B	0.4606	−0.5090	0.1576	0.046*	
C22	0.6160 (6)	−0.4773 (6)	0.1896 (3)	0.0377 (16)	
H22A	0.6761	−0.4352	0.1706	0.045*	
H22B	0.6469	−0.5509	0.2094	0.045*	
C23	0.5727 (5)	−0.3885 (5)	0.2345 (3)	0.0248 (12)	
H23A	0.6348	−0.3580	0.2580	0.030*	
H23B	0.5251	−0.4361	0.2595	0.030*	
C24	0.5088 (5)	−0.2753 (5)	0.2112 (2)	0.0229 (11)	
H24A	0.4407	−0.3032	0.1919	0.027*	
H24B	0.4900	−0.2194	0.2426	0.027*	
C25	0.6372 (4)	0.0087 (5)	0.0709 (2)	0.0174 (10)	
H25	0.665 (5)	−0.068 (6)	0.052 (3)	0.021*	
C26	0.6671 (4)	0.0212 (5)	0.1290 (2)	0.0173 (10)	
H26	0.714 (5)	−0.046 (6)	0.143 (3)	0.021*	
C27	0.6759 (4)	0.1413 (5)	0.1608 (2)	0.0207 (11)	
H27A	0.6663	0.1242	0.2015	0.025*	
H27B	0.6158	0.1963	0.1478	0.025*	
C28	0.7842 (5)	0.2112 (5)	0.1542 (2)	0.0232 (11)	
H28A	0.7878	0.2791	0.1824	0.028*	
H28B	0.8442	0.1530	0.1637	0.028*	
C29	0.8044 (5)	0.2672 (5)	0.0956 (3)	0.0261 (12)	
H29A	0.7412	0.3192	0.0844	0.031*	
H29B	0.8683	0.3226	0.0990	0.031*	
C30	0.8236 (5)	0.1735 (6)	0.0481 (2)	0.0252 (12)	
H30A	0.8426	0.0925	0.0656	0.030*	
H30B	0.8870	0.2015	0.0268	0.030*	
C31	0.7280 (4)	0.1540 (6)	0.0061 (2)	0.0227 (11)	
H31A	0.7174	0.2311	−0.0162	0.027*	
H31B	0.7474	0.0873	−0.0206	0.027*	
C32	0.6204 (4)	0.1200 (5)	0.0324 (2)	0.0192 (10)	
H32A	0.5934	0.1911	0.0543	0.023*	
H32B	0.5658	0.1001	0.0022	0.023*	

### Atomic displacement parameters (Å^2^)

**Table:**

	*U*^11^	*U*^22^	*U*^33^	*U*^12^	*U*^13^	*U*^23^
Ir1	0.01035 (9)	0.01310 (9)	0.01811 (10)	0.00069 (7)	0.00157 (7)	0.00070 (7)
Ir2	0.01052 (9)	0.01305 (9)	0.01793 (10)	0.00124 (7)	0.00105 (7)	0.00185 (7)
Cl1	0.0112 (6)	0.0164 (6)	0.0395 (8)	0.0024 (4)	0.0046 (5)	0.0088 (5)
Cl2	0.0111 (5)	0.0150 (5)	0.0308 (7)	0.0018 (4)	0.0035 (5)	0.0038 (5)
C1	0.013 (3)	0.034 (3)	0.026 (3)	−0.004 (2)	−0.002 (2)	0.007 (2)
C2	0.011 (2)	0.016 (2)	0.031 (3)	−0.0025 (19)	0.009 (2)	0.002 (2)
C3	0.024 (3)	0.034 (3)	0.025 (3)	−0.013 (2)	−0.001 (2)	0.006 (2)
C4	0.022 (3)	0.039 (3)	0.025 (3)	−0.009 (3)	0.004 (2)	0.007 (2)
C5	0.021 (3)	0.034 (3)	0.030 (3)	−0.002 (2)	0.004 (2)	0.007 (2)
C6	0.024 (3)	0.029 (3)	0.060 (5)	−0.011 (3)	0.010 (3)	−0.003 (3)
C7	0.025 (3)	0.047 (4)	0.054 (5)	−0.021 (3)	0.008 (3)	−0.020 (3)
C8	0.032 (4)	0.054 (4)	0.038 (4)	−0.026 (3)	0.016 (3)	−0.027 (3)
C9	0.017 (3)	0.022 (3)	0.027 (3)	0.005 (2)	0.001 (2)	0.007 (2)
C10	0.016 (3)	0.017 (3)	0.033 (3)	0.004 (2)	0.005 (2)	0.008 (2)
C11	0.023 (3)	0.016 (3)	0.047 (4)	−0.007 (2)	0.008 (3)	−0.005 (2)
C12	0.042 (4)	0.017 (3)	0.051 (4)	−0.001 (3)	0.019 (3)	−0.002 (3)
C13	0.053 (5)	0.028 (4)	0.121 (9)	0.019 (4)	0.020 (5)	0.025 (5)
C14	0.082 (7)	0.045 (5)	0.072 (6)	0.002 (5)	−0.024 (5)	0.021 (4)
C15	0.041 (4)	0.039 (4)	0.034 (4)	0.006 (3)	−0.003 (3)	0.018 (3)
C16	0.033 (4)	0.037 (3)	0.028 (3)	0.013 (3)	0.004 (3)	0.014 (3)
C17	0.012 (2)	0.016 (2)	0.025 (3)	0.0028 (19)	−0.004 (2)	0.0051 (19)
C18	0.017 (3)	0.017 (2)	0.025 (3)	0.002 (2)	0.001 (2)	0.008 (2)
C19	0.026 (3)	0.016 (2)	0.024 (3)	0.004 (2)	−0.002 (2)	−0.002 (2)
C20	0.042 (4)	0.023 (3)	0.032 (3)	0.010 (3)	−0.001 (3)	−0.005 (2)
C21	0.042 (4)	0.017 (3)	0.056 (5)	0.000 (3)	−0.008 (3)	0.002 (3)
C22	0.041 (4)	0.022 (3)	0.050 (4)	0.005 (3)	−0.013 (3)	0.006 (3)
C23	0.020 (3)	0.023 (3)	0.032 (3)	0.003 (2)	−0.002 (2)	0.011 (2)
C24	0.023 (3)	0.023 (3)	0.023 (3)	0.004 (2)	0.003 (2)	0.008 (2)
C25	0.011 (2)	0.019 (2)	0.022 (3)	−0.0007 (19)	0.0032 (19)	0.000 (2)
C26	0.011 (2)	0.015 (2)	0.026 (3)	0.0004 (19)	−0.0008 (19)	0.003 (2)
C27	0.015 (3)	0.022 (3)	0.024 (3)	0.000 (2)	0.002 (2)	−0.002 (2)
C28	0.021 (3)	0.021 (3)	0.027 (3)	−0.003 (2)	−0.002 (2)	−0.002 (2)
C29	0.019 (3)	0.025 (3)	0.034 (3)	−0.007 (2)	−0.002 (2)	0.005 (2)
C30	0.016 (3)	0.031 (3)	0.028 (3)	−0.006 (2)	0.001 (2)	0.008 (2)
C31	0.016 (3)	0.032 (3)	0.020 (3)	−0.005 (2)	0.002 (2)	0.008 (2)
C32	0.016 (3)	0.019 (2)	0.023 (3)	−0.0026 (19)	−0.001 (2)	0.002 (2)

### Geometric parameters (Å, °)

**Table:**

Ir1—C10	2.113 (5)	C14—H14B	0.990
Ir1—C2	2.123 (5)	C15—C16	1.536 (8)
Ir1—C1	2.138 (6)	C15—H15A	0.990
Ir1—C9	2.139 (5)	C15—H15B	0.990
Ir1—Cl1	2.3980 (12)	C16—H16A	0.990
Ir1—Cl2	2.4188 (12)	C16—H16B	0.990
Ir1—Ir2	3.7254 (3)	C17—C18	1.420 (8)
Ir2—C26	2.117 (5)	C17—C24	1.499 (7)
Ir2—C18	2.117 (5)	C17—H17	0.91 (7)
Ir2—C25	2.139 (5)	C18—C19	1.512 (8)
Ir2—C17	2.153 (5)	C18—H18	0.88 (7)
Ir2—Cl1	2.4036 (12)	C19—C20	1.541 (8)
Ir2—Cl2	2.4203 (12)	C19—H19A	0.990
C1—C2	1.395 (8)	C19—H19B	0.990
C1—C8	1.512 (9)	C20—C21	1.529 (9)
C1—H1	0.94 (2)	C20—H20A	0.990
C2—C3	1.512 (8)	C20—H20B	0.990
C2—H2	0.96 (2)	C21—C22	1.531 (9)
C3—C4	1.530 (8)	C21—H21A	0.990
C3—H3A	0.990	C21—H21B	0.990
C3—H3B	0.990	C22—C23	1.536 (9)
C4—C5	1.535 (8)	C22—H22A	0.990
C4—H4A	0.990	C22—H22B	0.990
C4—H4B	0.990	C23—C24	1.545 (7)
C5—C6	1.511 (9)	C23—H23A	0.990
C5—H5A	0.990	C23—H23B	0.990
C5—H5B	0.990	C24—H24A	0.990
C6—C7	1.531 (10)	C24—H24B	0.990
C6—H6A	0.990	C25—C26	1.418 (8)
C6—H6B	0.990	C25—C32	1.515 (7)
C7—C8	1.541 (8)	C25—H25	1.01 (6)
C7—H7A	0.990	C26—C27	1.499 (7)
C7—H7B	0.990	C26—H26	0.98 (6)
C8—H8A	0.990	C27—C28	1.546 (7)
C8—H8B	0.990	C27—H27A	0.990
C9—C10	1.414 (8)	C27—H27B	0.990
C9—C16	1.506 (8)	C28—C29	1.539 (8)
C9—H9	0.98 (7)	C28—H28A	0.990
C10—C11	1.516 (8)	C28—H28B	0.990
C10—H10	0.92 (7)	C29—C30	1.533 (9)
C11—C12	1.557 (8)	C29—H29A	0.990
C11—H11A	0.990	C29—H29B	0.990
C11—H11B	0.990	C30—C31	1.534 (8)
C12—C13	1.594 (11)	C30—H30A	0.990
C12—H12A	0.990	C30—H30B	0.990
C12—H12B	0.990	C31—C32	1.527 (7)
C13—C14	1.449 (11)	C31—H31A	0.990
C13—H13A	0.990	C31—H31B	0.990
C13—H13B	0.990	C32—H32A	0.990
C14—C15	1.480 (12)	C32—H32B	0.990
C14—H14A	0.990		
			
C10—Ir1—C2	86.1 (2)	C15—C14—H14A	108.1
C10—Ir1—C1	98.0 (2)	C13—C14—H14B	108.1
C2—Ir1—C1	38.2 (2)	C15—C14—H14B	108.1
C10—Ir1—C9	38.9 (2)	H14A—C14—H14B	107.3
C2—Ir1—C9	98.5 (2)	C14—C15—C16	114.9 (6)
C1—Ir1—C9	85.4 (2)	C14—C15—H15A	108.5
C10—Ir1—Cl1	93.30 (16)	C16—C15—H15A	108.5
C2—Ir1—Cl1	158.83 (16)	C14—C15—H15B	108.5
C1—Ir1—Cl1	161.11 (17)	C16—C15—H15B	108.5
C9—Ir1—Cl1	94.08 (16)	H15A—C15—H15B	107.5
C10—Ir1—Cl2	159.49 (17)	C9—C16—C15	111.8 (5)
C2—Ir1—Cl2	94.51 (15)	C9—C16—H16A	109.3
C1—Ir1—Cl2	95.00 (17)	C15—C16—H16A	109.3
C9—Ir1—Cl2	159.14 (16)	C9—C16—H16B	109.3
Cl1—Ir1—Cl2	78.84 (4)	C15—C16—H16B	109.3
C26—Ir2—C18	89.9 (2)	H16A—C16—H16B	107.9
C26—Ir2—C25	38.9 (2)	C18—C17—C24	123.2 (5)
C18—Ir2—C25	85.5 (2)	C18—C17—Ir2	69.2 (3)
C26—Ir2—C17	84.9 (2)	C24—C17—Ir2	119.2 (4)
C18—Ir2—C17	38.8 (2)	C18—C17—H17	115 (4)
C25—Ir2—C17	105.0 (2)	C24—C17—H17	115 (4)
C26—Ir2—Cl1	99.00 (14)	Ir2—C17—H17	104 (4)
C18—Ir2—Cl1	158.57 (16)	C17—C18—C19	124.2 (5)
C25—Ir2—Cl1	89.26 (14)	C17—C18—Ir2	71.9 (3)
C17—Ir2—Cl1	160.74 (15)	C19—C18—Ir2	115.1 (4)
C26—Ir2—Cl2	159.24 (15)	C17—C18—H18	118 (4)
C18—Ir2—Cl2	99.55 (15)	C19—C18—H18	113 (4)
C25—Ir2—Cl2	159.37 (15)	Ir2—C18—H18	106 (4)
C17—Ir2—Cl2	90.91 (15)	C18—C19—C20	114.1 (5)
Cl1—Ir2—Cl2	78.70 (4)	C18—C19—H19A	108.7
Ir1—Cl1—Ir2	101.77 (5)	C20—C19—H19A	108.7
Ir1—Cl2—Ir2	100.69 (4)	C18—C19—H19B	108.7
C2—C1—C8	124.3 (6)	C20—C19—H19B	108.7
C2—C1—Ir1	70.3 (3)	H19A—C19—H19B	107.6
C8—C1—Ir1	117.4 (4)	C21—C20—C19	114.8 (5)
C2—C1—H1	123 (4)	C21—C20—H20A	108.6
C8—C1—H1	106 (4)	C19—C20—H20A	108.6
Ir1—C1—H1	111 (4)	C21—C20—H20B	108.6
C1—C2—C3	122.6 (5)	C19—C20—H20B	108.6
C1—C2—Ir1	71.5 (3)	H20A—C20—H20B	107.5
C3—C2—Ir1	118.4 (4)	C20—C21—C22	116.0 (6)
C1—C2—H2	116 (4)	C20—C21—H21A	108.3
C3—C2—H2	111 (4)	C22—C21—H21A	108.3
Ir1—C2—H2	111 (4)	C20—C21—H21B	108.3
C2—C3—C4	110.5 (5)	C22—C21—H21B	108.3
C2—C3—H3A	109.6	H21A—C21—H21B	107.4
C4—C3—H3A	109.6	C21—C22—C23	116.9 (6)
C2—C3—H3B	109.6	C21—C22—H22A	108.1
C4—C3—H3B	109.6	C23—C22—H22A	108.1
H3A—C3—H3B	108.1	C21—C22—H22B	108.1
C3—C4—C5	115.7 (5)	C23—C22—H22B	108.1
C3—C4—H4A	108.4	H22A—C22—H22B	107.3
C5—C4—H4A	108.4	C22—C23—C24	115.5 (5)
C3—C4—H4B	108.4	C22—C23—H23A	108.4
C5—C4—H4B	108.4	C24—C23—H23A	108.4
H4A—C4—H4B	107.4	C22—C23—H23B	108.4
C6—C5—C4	115.6 (5)	C24—C23—H23B	108.4
C6—C5—H5A	108.4	H23A—C23—H23B	107.5
C4—C5—H5A	108.4	C17—C24—C23	108.7 (5)
C6—C5—H5B	108.4	C17—C24—H24A	110.0
C4—C5—H5B	108.4	C23—C24—H24A	110.0
H5A—C5—H5B	107.4	C17—C24—H24B	110.0
C5—C6—C7	116.8 (6)	C23—C24—H24B	110.0
C5—C6—H6A	108.1	H24A—C24—H24B	108.3
C7—C6—H6A	108.1	C26—C25—C32	122.2 (5)
C5—C6—H6B	108.1	C26—C25—Ir2	69.7 (3)
C7—C6—H6B	108.1	C32—C25—Ir2	120.5 (4)
H6A—C6—H6B	107.3	C26—C25—H25	116 (4)
C6—C7—C8	115.7 (6)	C32—C25—H25	115 (4)
C6—C7—H7A	108.3	Ir2—C25—H25	105 (3)
C8—C7—H7A	108.3	C25—C26—C27	125.6 (5)
C6—C7—H7B	108.3	C25—C26—Ir2	71.4 (3)
C8—C7—H7B	108.3	C27—C26—Ir2	115.1 (4)
H7A—C7—H7B	107.4	C25—C26—H26	113 (4)
C1—C8—C7	112.6 (6)	C27—C26—H26	116 (4)
C1—C8—H8A	109.1	Ir2—C26—H26	106 (4)
C7—C8—H8A	109.1	C26—C27—C28	114.7 (4)
C1—C8—H8B	109.1	C26—C27—H27A	108.6
C7—C8—H8B	109.1	C28—C27—H27A	108.6
H8A—C8—H8B	107.8	C26—C27—H27B	108.6
C10—C9—C16	124.5 (6)	C28—C27—H27B	108.6
C10—C9—Ir1	69.6 (3)	H27A—C27—H27B	107.6
C16—C9—Ir1	117.5 (4)	C29—C28—C27	116.5 (5)
C10—C9—H9	113 (4)	C29—C28—H28A	108.2
C16—C9—H9	113 (4)	C27—C28—H28A	108.2
Ir1—C9—H9	112 (4)	C29—C28—H28B	108.2
C9—C10—C11	122.4 (5)	C27—C28—H28B	108.2
C9—C10—Ir1	71.6 (3)	H28A—C28—H28B	107.3
C11—C10—Ir1	118.0 (4)	C30—C29—C28	115.7 (5)
C9—C10—H10	113 (4)	C30—C29—H29A	108.4
C11—C10—H10	114 (4)	C28—C29—H29A	108.4
Ir1—C10—H10	111 (4)	C30—C29—H29B	108.4
C10—C11—C12	111.0 (5)	C28—C29—H29B	108.4
C10—C11—H11A	109.4	H29A—C29—H29B	107.4
C12—C11—H11A	109.4	C29—C30—C31	115.5 (5)
C10—C11—H11B	109.4	C29—C30—H30A	108.4
C12—C11—H11B	109.4	C31—C30—H30A	108.4
H11A—C11—H11B	108.0	C29—C30—H30B	108.4
C11—C12—C13	114.9 (5)	C31—C30—H30B	108.4
C11—C12—H12A	108.5	H30A—C30—H30B	107.5
C13—C12—H12A	108.5	C32—C31—C30	115.5 (5)
C11—C12—H12B	108.5	C32—C31—H31A	108.4
C13—C12—H12B	108.5	C30—C31—H31A	108.4
H12A—C12—H12B	107.5	C32—C31—H31B	108.4
C14—C13—C12	116.7 (7)	C30—C31—H31B	108.4
C14—C13—H13A	108.1	H31A—C31—H31B	107.5
C12—C13—H13A	108.1	C25—C32—C31	109.3 (4)
C14—C13—H13B	108.1	C25—C32—H32A	109.8
C12—C13—H13B	108.1	C31—C32—H32A	109.8
H13A—C13—H13B	107.3	C25—C32—H32B	109.8
C13—C14—C15	116.9 (8)	C31—C32—H32B	109.8
C13—C14—H14A	108.1	H32A—C32—H32B	108.3
			
C10—Ir1—Ir2—C26	4.6 (3)	C1—Ir1—C9—C16	−132.1 (5)
C2—Ir1—Ir2—C26	−122.4 (3)	Cl1—Ir1—C9—C16	29.0 (5)
C1—Ir1—Ir2—C26	−174.4 (3)	Cl2—Ir1—C9—C16	−40.1 (8)
C9—Ir1—Ir2—C26	56.2 (3)	Ir2—Ir1—C9—C16	13.0 (6)
Cl1—Ir1—Ir2—C26	30.4 (2)	C16—C9—C10—C11	2.1 (8)
Cl2—Ir1—Ir2—C26	−150.3 (2)	Ir1—C9—C10—C11	112.0 (5)
C10—Ir1—Ir2—C18	−174.3 (3)	C16—C9—C10—Ir1	−110.0 (5)
C2—Ir1—Ir2—C18	58.6 (3)	C2—Ir1—C10—C9	−108.8 (3)
C1—Ir1—Ir2—C18	6.7 (3)	C1—Ir1—C10—C9	−72.4 (4)
C9—Ir1—Ir2—C18	−122.8 (3)	Cl1—Ir1—C10—C9	92.4 (3)
Cl1—Ir1—Ir2—C18	−148.6 (2)	Cl2—Ir1—C10—C9	158.9 (4)
Cl2—Ir1—Ir2—C18	30.8 (2)	Ir2—Ir1—C10—C9	108.4 (3)
C10—Ir1—Ir2—C25	−46.3 (3)	C2—Ir1—C10—C11	133.6 (5)
C2—Ir1—Ir2—C25	−173.4 (3)	C1—Ir1—C10—C11	170.0 (5)
C1—Ir1—Ir2—C25	134.7 (3)	C9—Ir1—C10—C11	−117.6 (6)
C9—Ir1—Ir2—C25	5.2 (3)	Cl1—Ir1—C10—C11	−25.2 (5)
Cl1—Ir1—Ir2—C25	−20.58 (19)	Cl2—Ir1—C10—C11	41.3 (8)
Cl2—Ir1—Ir2—C25	158.75 (18)	Ir2—Ir1—C10—C11	−9.2 (6)
C10—Ir1—Ir2—C17	133.3 (3)	C9—C10—C11—C12	86.9 (7)
C2—Ir1—Ir2—C17	6.2 (3)	Ir1—C10—C11—C12	172.0 (4)
C1—Ir1—Ir2—C17	−45.7 (3)	C10—C11—C12—C13	−45.3 (8)
C9—Ir1—Ir2—C17	−175.2 (3)	C11—C12—C13—C14	−59.2 (9)
Cl1—Ir1—Ir2—C17	159.0 (2)	C12—C13—C14—C15	104.1 (9)
Cl2—Ir1—Ir2—C17	−21.6 (2)	C13—C14—C15—C16	−72.8 (10)
C10—Ir1—Ir2—Cl1	−25.7 (2)	C10—C9—C16—C15	−86.5 (7)
C2—Ir1—Ir2—Cl1	−152.8 (2)	Ir1—C9—C16—C15	−169.6 (5)
C1—Ir1—Ir2—Cl1	155.3 (2)	C14—C15—C16—C9	77.0 (9)
C9—Ir1—Ir2—Cl1	25.8 (2)	C26—Ir2—C17—C18	−96.2 (3)
Cl2—Ir1—Ir2—Cl1	179.32 (8)	C25—Ir2—C17—C18	−62.5 (3)
C10—Ir1—Ir2—Cl2	154.9 (2)	Cl1—Ir2—C17—C18	160.9 (4)
C2—Ir1—Ir2—Cl2	27.9 (2)	Cl2—Ir2—C17—C18	104.2 (3)
C1—Ir1—Ir2—Cl2	−24.0 (2)	Ir1—Ir2—C17—C18	117.8 (3)
C9—Ir1—Ir2—Cl2	−153.5 (2)	C26—Ir2—C17—C24	146.5 (5)
Cl1—Ir1—Ir2—Cl2	−179.32 (8)	C18—Ir2—C17—C24	−117.3 (6)
C10—Ir1—Cl1—Ir2	160.45 (17)	C25—Ir2—C17—C24	−179.9 (4)
C2—Ir1—Cl1—Ir2	72.9 (4)	Cl1—Ir2—C17—C24	43.6 (7)
C1—Ir1—Cl1—Ir2	−72.8 (5)	Cl2—Ir2—C17—C24	−13.2 (4)
C9—Ir1—Cl1—Ir2	−160.62 (17)	Ir1—Ir2—C17—C24	0.5 (5)
Cl2—Ir1—Cl1—Ir2	−0.44 (5)	C24—C17—C18—C19	3.6 (8)
C26—Ir2—Cl1—Ir1	−158.60 (15)	Ir2—C17—C18—C19	−108.5 (5)
C18—Ir2—Cl1—Ir1	87.8 (4)	C24—C17—C18—Ir2	112.0 (5)
C25—Ir2—Cl1—Ir1	163.58 (15)	C26—Ir2—C18—C17	82.0 (3)
C17—Ir2—Cl1—Ir1	−58.1 (5)	C25—Ir2—C18—C17	120.7 (3)
Cl2—Ir2—Cl1—Ir1	0.44 (5)	Cl1—Ir2—C18—C17	−162.8 (3)
C10—Ir1—Cl2—Ir2	−68.5 (4)	Cl2—Ir2—C18—C17	−79.4 (3)
C2—Ir1—Cl2—Ir2	−159.26 (16)	Ir1—Ir2—C18—C17	−98.8 (3)
C1—Ir1—Cl2—Ir2	162.39 (17)	C26—Ir2—C18—C19	−158.0 (4)
C9—Ir1—Cl2—Ir2	72.1 (5)	C25—Ir2—C18—C19	−119.3 (4)
Cl1—Ir1—Cl2—Ir2	0.44 (5)	C17—Ir2—C18—C19	120.0 (5)
C26—Ir2—Cl2—Ir1	85.2 (4)	Cl1—Ir2—C18—C19	−42.8 (7)
C18—Ir2—Cl2—Ir1	−158.71 (16)	Cl2—Ir2—C18—C19	40.6 (4)
C25—Ir2—Cl2—Ir1	−55.8 (4)	Ir1—Ir2—C18—C19	21.3 (5)
C17—Ir2—Cl2—Ir1	163.23 (15)	C17—C18—C19—C20	−83.5 (7)
Cl1—Ir2—Cl2—Ir1	−0.43 (5)	Ir2—C18—C19—C20	−168.0 (4)
C10—Ir1—C1—C2	−73.1 (4)	C18—C19—C20—C21	73.4 (7)
C9—Ir1—C1—C2	−110.0 (4)	C19—C20—C21—C22	−72.6 (8)
Cl1—Ir1—C1—C2	160.8 (4)	C20—C21—C22—C23	103.7 (7)
Cl2—Ir1—C1—C2	90.9 (3)	C21—C22—C23—C24	−50.8 (8)
Ir2—Ir1—C1—C2	106.1 (3)	C18—C17—C24—C23	90.5 (6)
C10—Ir1—C1—C8	167.7 (5)	Ir2—C17—C24—C23	173.7 (4)
C2—Ir1—C1—C8	−119.2 (6)	C22—C23—C24—C17	−54.3 (7)
C9—Ir1—C1—C8	130.8 (5)	C18—Ir2—C25—C26	−95.4 (3)
Cl1—Ir1—C1—C8	41.6 (8)	C17—Ir2—C25—C26	−61.5 (3)
Cl2—Ir1—C1—C8	−28.3 (5)	Cl1—Ir2—C25—C26	105.4 (3)
Ir2—Ir1—C1—C8	−13.1 (6)	Cl2—Ir2—C25—C26	159.2 (3)
C8—C1—C2—C3	−2.1 (9)	Ir1—Ir2—C25—C26	118.2 (3)
Ir1—C1—C2—C3	−112.4 (5)	C26—Ir2—C25—C32	−116.2 (5)
C8—C1—C2—Ir1	110.3 (6)	C18—Ir2—C25—C32	148.3 (4)
C10—Ir1—C2—C1	108.2 (4)	C17—Ir2—C25—C32	−177.7 (4)
C9—Ir1—C2—C1	71.3 (4)	Cl1—Ir2—C25—C32	−10.9 (4)
Cl1—Ir1—C2—C1	−162.9 (3)	Cl2—Ir2—C25—C32	43.0 (7)
Cl2—Ir1—C2—C1	−92.4 (3)	Ir1—Ir2—C25—C32	1.9 (5)
Ir2—Ir1—C2—C1	−109.8 (3)	C32—C25—C26—C27	5.9 (8)
C10—Ir1—C2—C3	−134.1 (5)	Ir2—C25—C26—C27	−108.0 (5)
C1—Ir1—C2—C3	117.7 (6)	C32—C25—C26—Ir2	114.0 (5)
C9—Ir1—C2—C3	−171.0 (4)	C18—Ir2—C26—C25	83.0 (3)
Cl1—Ir1—C2—C3	−45.1 (7)	C17—Ir2—C26—C25	121.5 (3)
Cl2—Ir1—C2—C3	25.4 (4)	Cl1—Ir2—C26—C25	−77.5 (3)
Ir2—Ir1—C2—C3	7.9 (5)	Cl2—Ir2—C26—C25	−159.3 (3)
C1—C2—C3—C4	−90.9 (7)	Ir1—Ir2—C26—C25	−96.3 (3)
Ir1—C2—C3—C4	−176.1 (4)	C18—Ir2—C26—C27	−155.7 (4)
C2—C3—C4—C5	49.5 (7)	C25—Ir2—C26—C27	121.3 (5)
C3—C4—C5—C6	55.4 (8)	C17—Ir2—C26—C27	−117.1 (4)
C4—C5—C6—C7	−102.3 (7)	Cl1—Ir2—C26—C27	43.9 (4)
C5—C6—C7—C8	71.4 (9)	Cl2—Ir2—C26—C27	−38.0 (7)
C2—C1—C8—C7	83.8 (8)	Ir1—Ir2—C26—C27	25.1 (5)
Ir1—C1—C8—C7	167.7 (5)	C25—C26—C27—C28	−82.6 (7)
C6—C7—C8—C1	−74.2 (9)	Ir2—C26—C27—C28	−166.9 (4)
C2—Ir1—C9—C10	72.7 (3)	C26—C27—C28—C29	69.1 (7)
C1—Ir1—C9—C10	108.7 (4)	C27—C28—C29—C30	−69.2 (7)
Cl1—Ir1—C9—C10	−90.2 (3)	C28—C29—C30—C31	104.2 (6)
Cl2—Ir1—C9—C10	−159.2 (4)	C29—C30—C31—C32	−53.8 (7)
Ir2—Ir1—C9—C10	−106.2 (3)	C26—C25—C32—C31	89.4 (6)
C10—Ir1—C9—C16	119.2 (6)	Ir2—C25—C32—C31	173.4 (4)
C2—Ir1—C9—C16	−168.1 (5)	C30—C31—C32—C25	−53.2 (6)
